# Nrf2–ARE Signaling Acts as Master Pathway for the Cellular Antioxidant Activity of Fisetin

**DOI:** 10.3390/molecules24040708

**Published:** 2019-02-15

**Authors:** Huihui Zhang, Wan Zheng, Xiangling Feng, Fei Yang, Hong Qin, Shusong Wu, De-Xing Hou, Jihua Chen

**Affiliations:** 1 Xiangya School of Public Health, Central South University, Changsha 410128, China; zhanghuihui182@foxmail.com (H.Z.); zhengwan0810@foxmail.com (W.Z.); fxl7375@163.com (X.F.); phfyang@csu.edu.cn (F.Y.); qinhong@csu.edu.cn (H.Q.); 2 1515 Core Research Program, Hunan Co-Innovation Center for Utilization of Botanical Functional Ingredients, Changsha 410128, China; wush688@126.com; 3 Course of Biological Science and Technology, The United Graduate School of Agricultural Sciences, Department of Food Science and Biotechnology, Faculty of Agriculture, Kagoshima University, Korimoto 1-21-24, Kagoshima 890-0065, Japan

**Keywords:** fisetin, oxidative stress, Nrf2–ARE pathway, HO-1

## Abstract

Fisetin, a dietary flavonoid, is reported to have cellular antioxidant activity with an unclear mechanism. In this study, we investigated the effect of fisetin on the nuclear factor, erythroid 2-like 2 (Nrf2) signaling pathway in HepG2 cells to explore the cellular antioxidant mechanism. Fisetin upregulated the mRNA expression of heme oxygenase-1 (HO-1), glutamate-cysteine ligase catalytic subunit (GCLC), glutamate-cysteine ligase modifier subunit (GCLM), and NAD(P)H quinone oxidoreductase-1 (NQO1), and induced the protein of HO-1 but had no significant effect on the protein of GCLC, GCLM and NQO1. Moreover, nuclear accumulation of Nrf2 was clearly observed by immunofluorescence analysis and western blotting after fisetin treatment, and an enhanced luciferase activity of antioxidant response element (ARE)-regulated transactivation was obtained by dual-luciferase reporter gene assays. In addition, fisetin upregulated the protein level of Nrf2 and downregulated the protein level of Kelch-like ECH-associated protein 1 (Keap1). However, fisetin had no significant effect on Nrf2 mRNA expression. When protein synthesis was inhibited with cycloheximide (CHX), fisetin prolonged the half-life of Nrf2 from 15 min to 45 min. When blocking Nrf2 degradation with proteasome inhibitor MG132, ubiquitinated proteins were enhanced, and fisetin reduced ubiquitination of Nrf2. Taken together, fisetin translocated Nrf2 into the nucleus and upregulated the expression of downstream HO-1 gene by inhibiting the degradation of Nrf2 at the post-transcriptional level. These data provide the molecular mechanism to understand the cellular antioxidant activity of fisetin.

## 1. Introduction

Reactive oxygen species (ROS) are chemical species containing oxygen, such as peroxides, superoxide, hydroxyl radical, singlet oxygen [1]. ROS are formed as a natural byproduct of the normal metabolism of oxygen and have important roles in cell signaling and homeostasis. However, during times of environmental stress (for instance, ultraviolet radiation or heat exposure), ROS levels can increase dramatically [2]. If excessive ROS are not removed effectively, this will cause oxidative stress including DNA damage, lipid peroxidation, or protein damage. Cumulatively, oxidative stress leads to pathological changes, and ROS are associated with the early onset of more than 200 clinical diseases [3], especially chronic diseases, such as Parkinson’s disease, Alzheimer’s disease, cancer, cardiovascular disease, diabetes, etc. [4,5,6,7,8].

Phase II enzymes play a key role in defending against oxidative damage and in scavenging excess ROS. Nuclear factor, erythroid 2-like 2 (Nrf2) is a key factor for regulating the expression of many antioxidant genes in cells. Under physiological conditions, Nrf2 is inactivated by binding to Kelch-like ECH-associated protein 1 (Keap1) in the cytoplasm. When exposed to reactive oxidants/electrophiles, Nrf2 dissociates from Keap1. The activated Nrf2 translocates into the nucleus and binds to antioxidant response element (ARE). ARE contains the consensus sequence 5′-TGANNNNGC-3′ [9], which is shared by the promotors of many phase II enzyme genes. The activated Nrf2–ARE pathway induces the expression of downstream phase II enzymes [10,11], such as glutathione S-transferase (GST), NAD(P)H quinone oxidoreductase-1 (NQO1), heme oxygenase-1 (HO-1), glutamate-cysteine ligase catalytic subunit (GCLC), and glutamate-cysteine ligase modifier subunit (GCLM). Phase II enzymes scavenge excess ROS in cells to counteract intracellular oxidative damage, which has positive effects for disease prevention and treatment.

A growing body of evidence indicates that flavonoids, such as curcumin, quercetin, and myricetin can maintain intracellular redox homeostasis attributed not only to the direct scavenging of intracellular ROS, but also to the upregulation of the intracellular antioxidant system [12,13,14]. Fisetin is a flavonoid that is widely found in fruits and vegetables [15]. Fisetin performs numerous biological activities, including antioxidant, anti-inflammatory, antitumor, antineurodegenerative activities, for the prevention or treatment of oxidative damage, inflammation processes, neurodegenerative disorders, and cancers [16,17,18,19,20]. It has been reported that fisetin protects against H_2_O_2_-induced cell damage by inhibiting ROS generation, thereby maintaining the protective role of the cellular glutathione (GSH) system [21]. So far, the antioxidant mechanism of fisetin has not been fully elucidated. In this study, we attempt to define the cellular antioxidant mechanism of fisetin by investigating how fisetin affects the Nrf2-mediated antioxidant pathway.

## 2. Results

### 2.1. Effect of Fisetin on Cell Viability

**Figure 1:**
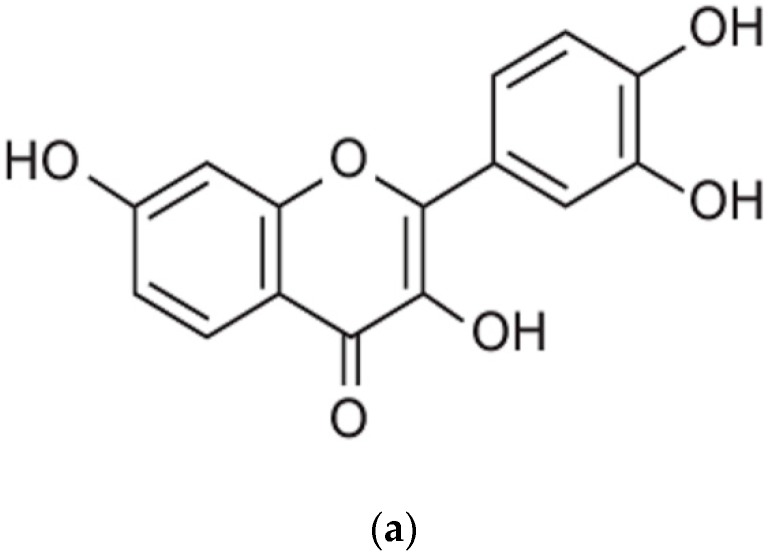

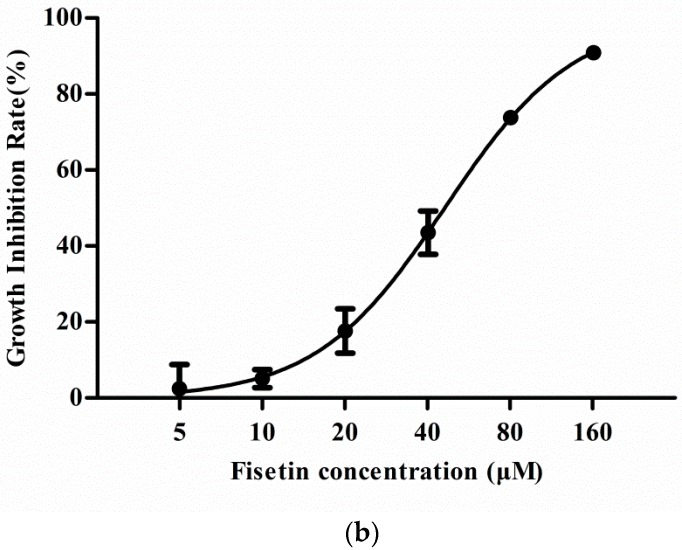
Effects of fisetin on HepG2 cell viability. (**a**) Chemical structure of fisetin; (**b**) HepG2 cells were incubated with different concentrations of fisetin (0, 5, 10, 20, 40, 80, 160 μM) for 24 h, and cell viability was detected by methyl thiazolyl tetrazolium (MTT) assay. DMSO treatment was used as a control. Data are presented as the mean ± SD of three independent experiments.

To investigate the antioxidant mechanism of fisetin (structure shown in Figure 1a) at the intracellular level, HepG2 cells were exposed to different concentrations of fisetin for 24 h, and a methyl thiazolyl tetrazolium (MTT) assay was subsequently performed. We found that the growth inhibition rate consistently increased as the concentration of fisetin increased (Figure 1b). Therefore, 10, 20, and 30 μM concentrations of fisetin were used for the experiments.

### 2.2. Effect of Fisetin on the Level of Phase II Enzyme

**Figure 2:**
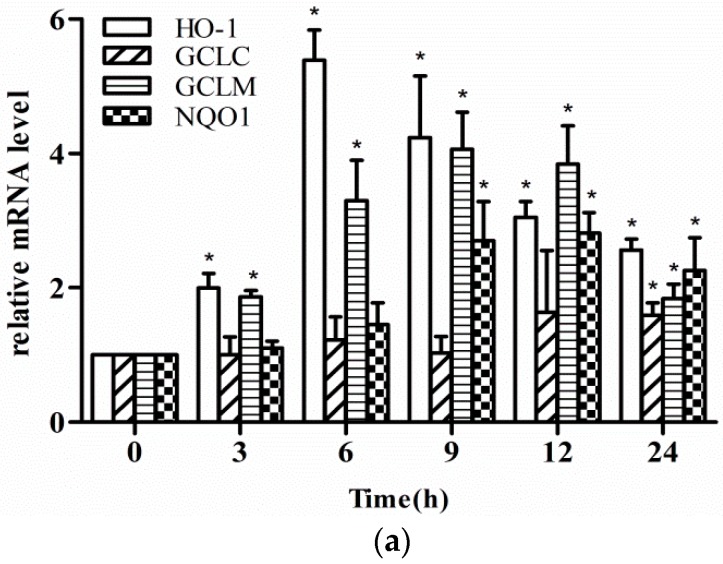

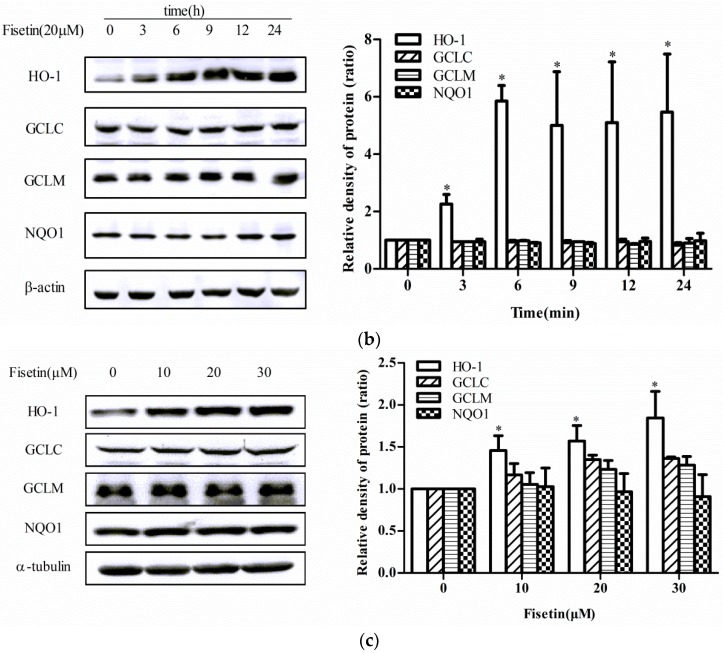
Effect of fisetin on phase II enzyme expression. (**a**) mRNA expression of heme oxygenase-1 (HO-1), glutamate-cysteine ligase catalytic subunit (GCLC), glutamate-cysteine ligase modifier subunit (GCLM), and NAD(P)H quinone oxidoreductase-1 (NQO1) treated with fisetin (20 μM) for 0 to 24 h; (**b**) protein level of HO-1, GCLC, GCLM, and NQO1 treated with fisetin (20 μM) for 0 to 24 h; (**c**) protein level of HO-1, GCLC, GCLM, and NQO1 treated with fisetin (0, 10, 20, 30 μM) for 6 h. Data represent mean ± SD of three independent experiments. * *P* < 0.05 vs. control group.

Since phase II enzymes can protect cells from oxidative stress, we hypothesized that fisetin may enhance the levels of phase II enzymes. Thus, we treated the cells with the indicated time and concentrations of fisetin and then detected the mRNA expression of typical phase II enzymes by qPCR and protein levels by western blotting analysis. As shown in Figure 2a, the mRNA expression of HO-1, GCLC, GCLM, and NQO1 was increased separately at different times after fisetin treatment. Figure 2b,c show that the protein level of HO-1 increased time-dependently and dose-dependently, however, the protein level of GCLC, GCLM, and NQO1 showed no significant changes.

### 2.3. Fisetin-Induced Nuclear Accumulation of Nrf2

**Figure 3:**
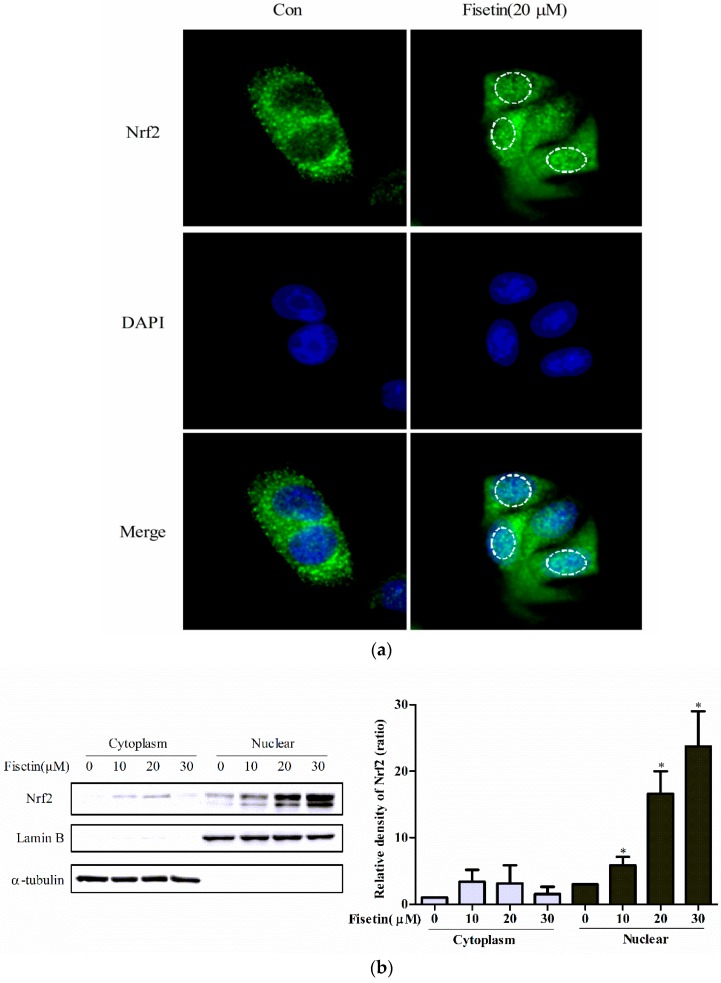
Effect of fisetin on nuclear accumulation of nuclear factor, erythroid 2-like 2 (Nrf2). (**a**) Effect of fisetin on Nrf2 translocalization into the nucleus performed with immunofluorescence analysis. HepG2 cells were treated with 20 μM fisetin for 6 h. The image shows Nrf2 (green)-stained Fluor-conjugated secondary antibody and the nucleus (blue) stained with DAPI, and the merged image of fisetin-treated cells shows the nuclear location of Nrf2 protein; (**b**) effect of fisetin on Nrf2 accumulation into the nucleus performed with western blot analysis. Cells were treated with fisetin (0, 10, 20, 30 μM) for 6 h and then nuclear and cytoplasmic proteins were extracted. Data represent mean ± SD of three independent experiments. * *P* < 0.05 vs. control group.

Under normal conditions, unactivated Nrf2 is rapidly degraded through the ubiquitin-26S proteasome pathway in cytoplasm. After the dissociation with Keap1, activated Nrf2 translocated into the nucleus and mediated phase II enzyme induction by binding to endogenous ARE. The results of immunofluorescence staining indicated that Nrf2 translocated into the nucleus (green) with fisetin treatment (Figure 3a). Western blot analysis further confirmed the translocation of Nrf2 into the nucleus after exposure to fisetin for 6 h (Figure 3b). 

### 2.4. Effect of Fisetin on the Transcriptional Activity of ARE-Regulated Luciferase Activity

**Figure 4:**
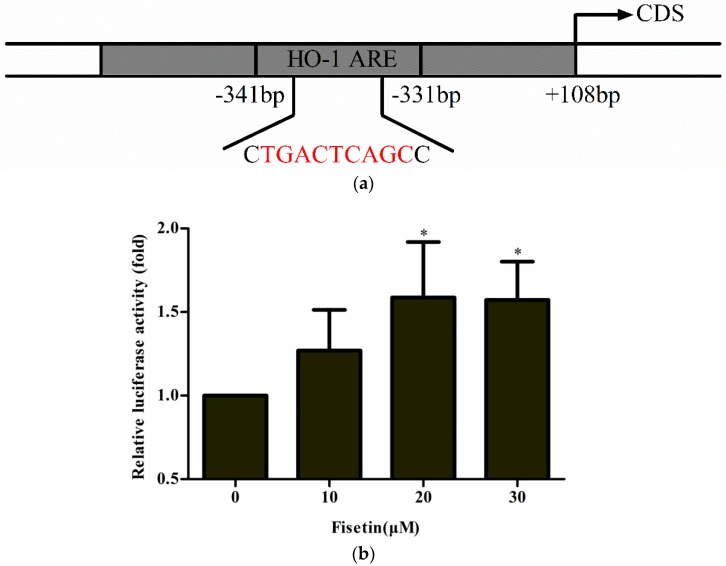
Effect of fisetin on the transcriptional activity of antioxidant response element (ARE)-regulated luciferase reporter gene. (**a**) The sequences of the binding site of Nrf2 and HO-1 ARE are given, and the colored sequences show the binding sequences of Nrf2 and ARE of pARE-luc; (**b**) ARE-regulated luciferase reporter gene activity in HepG2. The ARE-luciferase vector was introduced into cells, and then cells were treated with or without several concentrations of fisetin for 24 h. Luciferase activity was measured with an analyzer enzyme fluorescent assay. Data represent mean ± SD of three independent experiments. * *P* < 0.05 vs. control group.

To clarify whether Nrf2 translocated into the nucleus was bound to ARE sites and caused the upregulation of downstream genes after treatment with fisetin, pARE-luc plasmid containing 2-tandem repeats of HO-1 ARE (Figure 4a) was transfected into HepG2 cells. Treatment with fisetin resulted in a significant increase in luciferase activity (Figure 4b). This result indicated that Nrf2 accumulated into the nucleus by fisetin might bind to ARE sites, thereby upregulating the expression of downstream HO-1 genes. 

### 2.5. Effect of Fisetin on Nrf2 Expression

**Figure 5:**
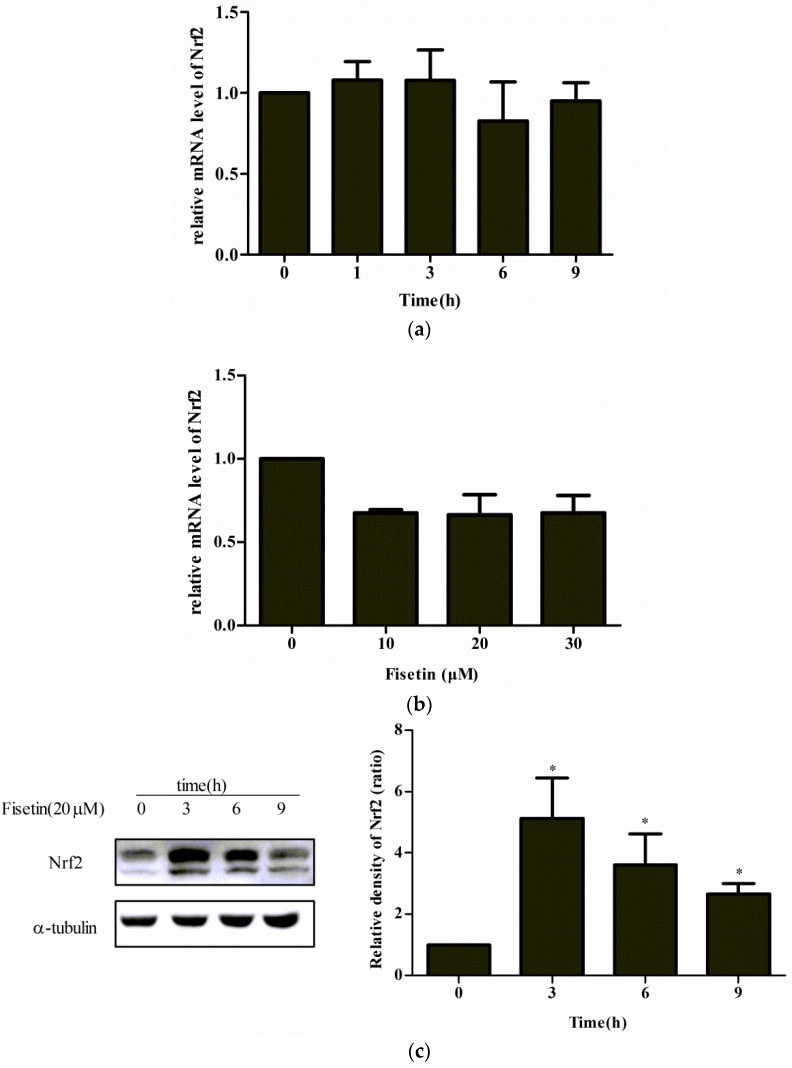

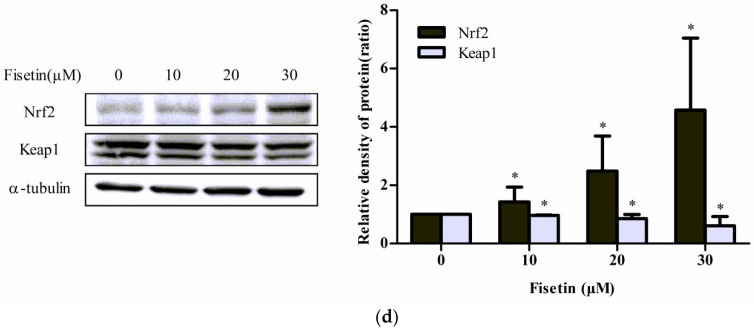
Effect of fisetin on Nrf2 expression. (**a**) mRNA expression of Nrf2 treated with fisetin (20 μM) for 0 to 9 h; (**b**) mRNA expression of Nrf2 treated with fisetin (0, 10, 20, 30 μM) for 6 h; (**c**) protein level of Nrf2 treated with fisetin (20 μM) for 0 to 9 h; (**d**) protein level of Nrf2 treated with fisetin (0, 10, 20, 30 μM) for 6 h. Data represent mean ± SD of three independent experiments. * *P* < 0.05 vs. control group.

Nrf2 level increased by fisetin is possibly regulated from a transcriptional or post-transcriptional level. To shed light on this issue, we examined the mRNA and protein level of Nrf2 after treatment with fisetin, using qPCR and western blotting analysis, respectively. As shown in Figure 5, no significant change in Nrf2 mRNA was detected (Figure 5a,b), but significantly increased Nrf2 protein and decreased Keap1 protein were detected after treatment with fisetin (Figure 5c,d). These data suggest that Nrf2 level increased by fisetin was regulated at the post-transcriptional level.

### 2.6. Fisetin Increased the Stability of Nrf2 Protein

**Figure 6:**
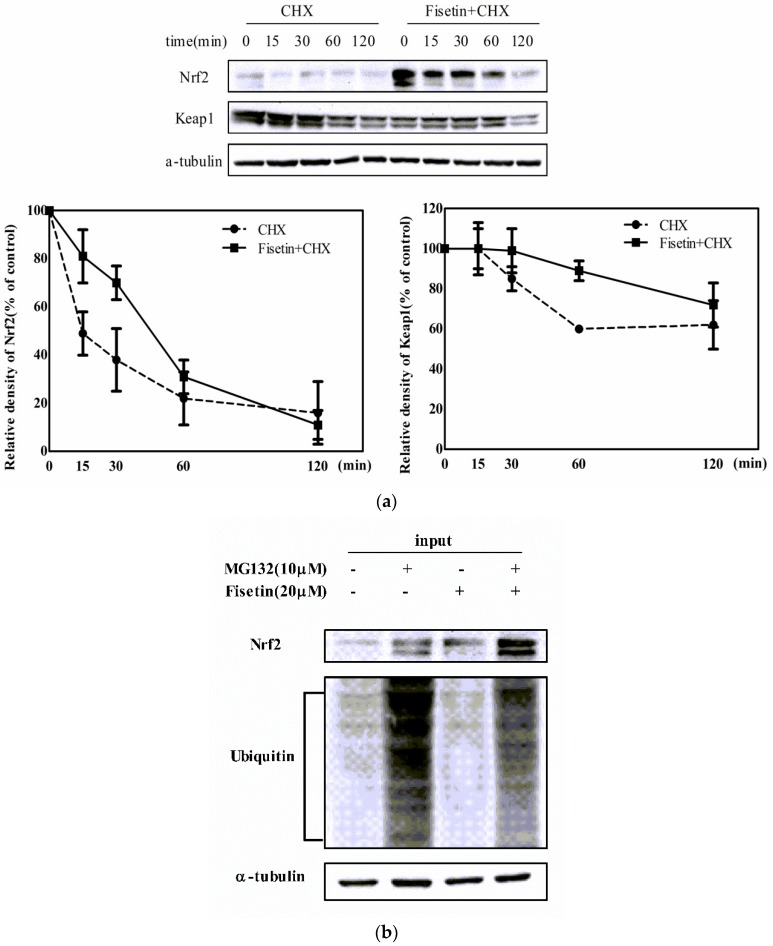

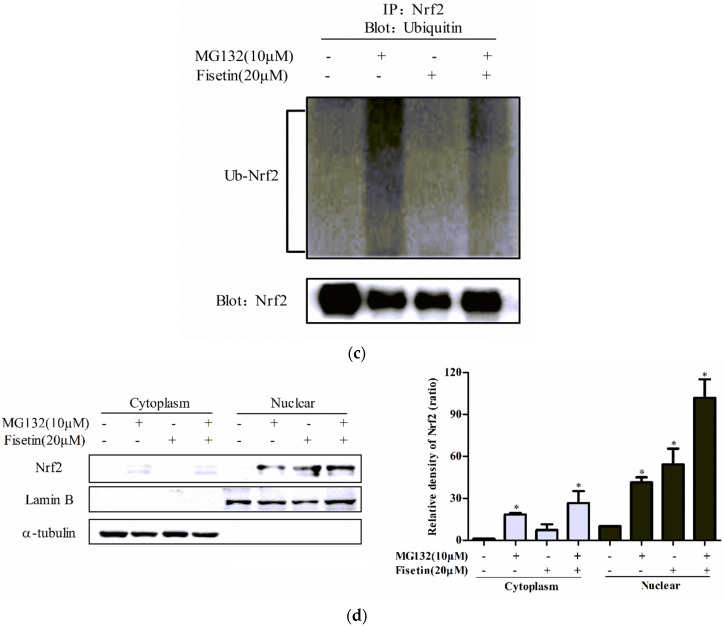
Effect of fisetin on the stability of Nrf2 protein. (**a**) Fisetin prolonged the half-life of Nrf2 protein. Cells were treated with or without fisetin (20 μM) for 4 h and then treated with cycloheximide (CHX, 5 μg/mL) at the indicated times (0, 15, 30, 60, 120 min); (**b**) western blot analysis of Nrf2 and ubiquitin. HepG2 cells were treated with or without MG132 (10 μM) for 1 h and then treated with or without fisetin (20 μM) for 6 h. Whole-cell lysates were used to detect Nrf2 and ubiquitin with their antibodies; (**c**) effects of fisetin on the ubiquitination of Nrf2. Cell lysates were immunoprecipitated with Nrf2 antibody, and precipitated proteins were analyzed by SDS−PAGE with the ubiquitin antibody; (**d**) fisetin promoted the migration of Nrf2 protein into the nucleus. HepG2 cells were treated with MG132 (10 μM) for 1 h and then treated with fisetin (20 μM) for 6 h, and nuclear and cytoplasmic proteins were extracted for western blot analysis. Data represent mean ± SD of three independent experiments. * *P* < 0.05 vs. control group.

Since fisetin has no effect on the transcription of Nrf2, we hypothesized that fisetin increased the protein stability of Nrf2 at the post-transcriptional level. Cells were treated with or without fisetin and cycloheximide (CHX), a protein synthesis inhibitor. As shown in Figure 6a, compared with the CHX group, Nrf2 was increased and Keap1 was decreased in the CHX + Fisetin group at the same timepoint. The half-life of Nrf2 protein in the CHX group was less than 15 min, while in the CHX + Fisetin group it was about 45 min. However, the half-life of Keap1 protein did not change significantly. This indicates that fisetin could prolong the half-life of Nrf2 protein, rather than stimulate Keap1 degradation.

Under physiological conditions, Nrf2 binds to Keap1 in the cytosol and is rapidly degraded by the 26S proteasome after ubiquitination. To determine whether the increased level of Nrf2 by fisetin was due to the suppression of Nrf2 ubiquitination, we examined the ubiquitination of Nrf2 by immunoprecipitation. As shown in Figure 6b, treatment with MG132 caused an increase in ubiquitinated protein that was reduced by cotreatment with fisetin. Meanwhile, the Nrf2 protein level was enhanced after treatment with MG132 or fisetin alone or in combination. After immunoprecipitation with anti-Nrf2 antibody, a significant reduction in the ubiquitination of Nrf2 was detected in the cells cotreated with fisetin and MG132 (Figure 6c). In addition, Nrf2 protein in the nucleus was higher than that in the cytoplasm in the same treatment group (Figure 6d). Taken together, these results demonstrate that fisetin inhibited the ubiquitylation degradation of Nrf2 and promoted the migration of Nrf2 into the nucleus to enhance gene transcription.

## 3. Discussion

Elevated oxidative stress is prevalent in patients with chronic kidney disease (CKD) and is associated with increased morbidity and mortality [22]. ROS, as intermediate product of oxidative stress, is associated with the early onset of many clinical diseases, especially chronic diseases. In diabetes, chronic and excessive ROS results in lower levels of insulin secretion and even insulin resistance by affecting the signaling pathway such as insulin or insulin-like growth factor (IGF)-1, insulin receptor (IR), insulin receptor substrate (IRS)-1, and phosphatidylinositol-3 kinase (PI3K)/Akt or ERK kinases [23].

Phytochemicals are being studied for the prevention and treatment of chronic diseases. Strawberry extracts not only improved lipid metabolism by decreasing triglycerides and lipoprotein (LDL)-cholesterol contents, but also improved the redox state of HepG2 cells by modulating antioxidant enzyme activity and ROS generation [24]. Fisetin, a flavonoid, has also been reported to possess antioxidant, neurotrophic properties, directly scavenging ROS or affecting signaling pathways [25,26]. Yen et al. reported that fisetin can scavenge ROS production by inducing Nrf2-driven oxidative stress response gene HO-1, GCLC, and GCLM mRNA expression so as to protect PC12 cells from tunicamycin-induced cytotoxicity [27]. In this study, fisetin treatment enhanced HO-1, GCLC, GCLM, and NQO1 mRNA expression (Figure 2a). Furthermore, we found that fisetin enhanced the protein level of HO-1 time-dependently and dose-dependently, but not GCLC, GCLM, and NQO1 (Figure 2b,c). The GCL enzyme is a dimer formed by GCLC and GCLM, and GCL catalyzes the formation of GSH, which is important in the oxidative stress defense. Some previous studies reported that fisetin could reduce oxidative stress by inducing both the mRNA and protein expression of GCLC, GCLM, and HO-1 in immortal mouse hippocampal HT22 cells [28]. However, Xiaomei Zheng found that the protein level of GCLM was not consistent with mRNA expression when exposed to diesel exhaust particles (DEPs), and this might be due to post-transcriptional regulatory mechanisms [29]. Interestingly, in macrophages, treatment with fisetin induced HO-1, GCLC, GST, and NQO1 mRNA expression as well as HO-1 protein level at the indicated times, but the protein of GCLC and NQO1 were not detected [30]. It is still unclear why fisetin can enhance the mRNA expression but not the protein expression of other antioxidant enzymes. This needs to be clarified in future work. 

Nrf2, a transcription factor activated by oxidative stress, acts to promote antioxidant enzymes whose promoters share the same ARE sequence. In a mouse model of traumatic brain injury (TBI), fisetin plays a critical role in neuroprotection partly through activation of the Nrf2–ARE pathway as well as Nrf2 downstream antioxidant enzymes such as HO-1 and NQO-1 [31]. In our results, we found that fisetin increased the protein level of Nrf2 at 3 h (maybe earlier than 3 h) and then gradually declined. HO-1 mRNA expression was activated at 3 h, consistent with the role of Nrf2 as an inducer of phase II enzymes. Combined with the time of increased HO-1 protein, we selected fisetin intervention for 6 h and Nrf2 increased dose-dependently (Figure 5c,d). Surprisingly, Nrf2 mRNA showed no significant changes at different doses and times after fisetin treatment (Figure 5a,b), suggesting that fisetin may induce the expression of Nrf2 at the post-transcriptional level but not at the transcriptional level. Some studies of other flavonoids have not been completely consistent with the results of our study. Quercetin not only increases the level of Nrf2 protein but also promotes the expression of Nrf2 mRNA in HepG2 cells [14]; tert-butylhydroquinone (tBHQ) increases Nrf2 protein at the transcriptional level but does not increase Nrf2 mRNA [32,33].

Under normal conditions, Nrf2 is repressed by Keap1, which is an adaptor protein for a Cullin 3 (Cul3)-dependent ubiquitination and degradation of Nrf2. During oxidative stress, Nrf2 is derepressed and activates the transcription of cytoprotective genes [34]. Nrf2 that entered into the nucleus binds to small Maf (sMaf) protein forming a heterodimer and then binds to the ARE on the promoter of the phase II enzyme, upregulating the expression of the phase II enzyme gene to resist oxidative damage [13,35,36,37,38,39]. As expected, immunofluorescence analysis confirmed the translocation of Nrf2 with fisetin treatment, and western blotting further demonstrated that fisetin promoted Nrf2 entry into the nucleus (Figure 3). In addition, dual luciferase reporter assays indicated that fisetin treatment increased the binding of Nrf2–ARE and ARE-luciferase reporter activity dose-dependently (Figure 4). The result was consistent with that obtained in another work where the effect of fisetin in human umbilical vein endothelial cells was investigated [40]. 

Under physiological conditions, Nrf2 binds to Keap1 in the cytoplasm and is rapidly degraded by the 26S proteasome before Cul3 ubiquitin E3 ligase polyubiquitination [41]. Studies have shown that the half-life of Nrf2 is less than 15 min [42]. When exposed to exogenous substances, thus leading to oxidative stress, Nrf2 dissociates from Keap1 and enters into the nucleus to protect from ubiquitination degradation. Arsenic stabilized Nrf2 protein, extending the half-life of Nrf2 from 21 to 200 min by inhibiting the Keap1 Cul3-dependent ubiquitination and proteasomal turnover of Nrf2 [33]. Li et al. [43] also found that the half-life of Nrf2 protein was prolonged from 14.7 min in untreated cells to 27.6 min in 7-O-methylbiochanin A (7-MBA)-treated cells. The literature supports the results of our study. In the present study, after treatment with the protein synthesis inhibitor CHX, fisetin prolonged the half-life of Nrf2 from 15 min to 45 min (Figure 6a), thereby increasing the stability of Nrf2 protein. Nrf2 is kept in the cytoplasm by Keap1, which controls the ubiquitination of Nrf2. To determine if the ubiquitination of Nrf2 is altered in fisetin-treated cells, we measured the levels of Nrf2 ubiquitination after exposure to fisetin. The data indicate that fisetin inhibited the ubiquitination degradation of Nrf2, resulting in increased nuclear translocation of Nrf2 (Figure 6b–d). Our data are in agreement with other findings. For example, withaferin A (WA) interfered with the Nrf2 activation pathway at an early step in the signaling cascade in the cytoplasm and decreased Nrf2 ubiquitination to maintain stability [44]. 6-Methylthiohexyl isothiocyanate (6-MTITC) modulated ARE-driven NQO1 expression by stabilizing Nrf2 with enhanced Keap1 modification and decreased Nrf2 ubiquitination degradation [45]. The above results show that fisetin can maintain Nrf2 stability by inhibiting its degradation.

## 4. Materials and Methods 

### 4.1. Reagents and Antibodies

Fisetin from Selleck (Houston, TX, USA) was dissolved in DMSO. Dimethyl sulfoxide (DMSO) and methyl thiazolyl tetrazolium (MTT) were purchased from Sigma–Aldrich (St. Louis, MO, USA). RPMI 1640 and fetal bovine serum (FBS) were purchased from Gibco (Grand Island, NY, USA). Penicillin–streptomycin solution (100×) and trypsin were purchased from Gen-View (Calimesa, CA, USA). pARE-luc plasmid, pGL6 plasmid, nuclear and cytoplasmic protein extraction kit, RIPA lysis buffer, cell lysis buffer for western blotting and IP, phenylmethyl sulfonylfluoride (PMSF) (100 mM), Protein A agarose (Fast Flow), dual-luciferase assay kit, and trizol reagent was obtained from Beyotime (Shanghai, China). Primary antibodies against Keap1, HO-1, NQO1, Lamin B, GCLC, GCLM, and Ub were purchased from Santa Cruz (Dallas, CA, USA). Rabbit IgG was purchased from Proteintech (Chicago, IL, USA). Primary antibodies against α-tubulin were obtained from ABclonal (Boston, MA, USA). Primary antibody against Nrf2 was obtained from Abcam (Cambridge, UK). Horseradish peroxidase (HRP)-conjugated goat anti-mouse IgG and HRP-conjugated goat anti-rabbit IgG (H + L) were purchased from Dingguo (Beijing, China). HRP-conjugated rabbit anti-goat IgG (H + L) was purchased from CWBIO (Beijing, China). Fluor-conjugated goat anti-rabbit IgG (H + L) was purchased from Thermo Fisher (St. Louis, MO, USA). All other reagents were analytical or biological grade.

### 4.2. Cell Culture and Treatment

HepG2 cells were obtained from the Cancer Research Institute of Central South University (Changsha, China) and cultured in RPMI 1640 medium supplemented with 10% FBS and penicillin–streptomycin solution in a humidified incubator at 37 °C and 5% CO_2_. Normally, HepG2 cells were seeded in 6 cm dish at 37 °C for 24 h, then fisetin stock solution was added directly to cell culture media for different times or at a final concentration of 10, 20, or 30 μM for 6 h.

### 4.3. MTT Assay

The MTT assay was used to assess the impact on cell viability. Cells were seeded in 96-well plates at a concentration of 5 × 10^3^ cells/well, cultured for 24 h and subsequently treated with different concentrations of fisetin (0, 5, 10, 20, 40, 80, and 160 μM) for 24 h. Subsequently, the culture medium was carefully removed from each well, 110 μL RPMI 1640 medium containing MTT (5 mg/mL) was added to each well, and cells were incubated for 4 h at 37 °C. The MTT solution was then discarded, and 150 μL DMSO was added into each well to dissolve formazan crystal at room temperature. Absorbance was measured using a microplate reader (BioTek, Winooski, VT, USA) at the wavelength of 490 nm. Cell viability was expressed as a percentage of MTT reduction. Each experiment was repeated 3 times.

### 4.4. Quantitative Real-Time PCR

Total RNA was extracted from treated cells using TRIzol reagent according to the manufacturer’s instructions. The purity (A260/A280 ratio) and concentration of RNA were obtained using an ultra-micro spectrophotometer (Implen, Munich, Germany), and RNA quality was confirmed by gel electrophoresis. Total RNA was used to synthesize cDNA utilizing an RNA reverse transcription kit (Thermo Fisher, MO, USA) according to the manufacturer’s instructions and then amplified with primer bases on HO-1, GCLC, GCLM, NQO1, Nrf2, and glyceraldehyde phosphate dehydrogenase (GAPDH) (internal control) sequences by quantitative real-time PCR (qPCR) assay using a LightCycler 96 Real-Time PCR System (Roche, Basel, Switzerland). Each 10 µL of reaction mixture contained 2× SYBR Green Master Mix (5 µL), forward primer (0.4 µL), reverse primer (0.4 µL), ddH2O (3.2 µL), and cDNA (1 µL). The PCR reaction protocol consisted of 45 cycles at 95 °C for 10 s, 60 °C for 10 s, and 72 °C for 45 s. The following primers were used in the present study: HO-1, F: 5′-CTGACCCATGACACCAAGGAC-3′, R: 5′-AAAGCCCTACAGCAACTGTCG-3′; GCLC, F: 5′-GGCACAAGGACGTTCTCAAGT-3′, R: 5′-CAGACAGGACCAACCGGAC-3′; GCLM, F: 5′-GGGAACCTGCTGAACTGG-3′, R: 5′-GCATGAGATACAGTGCATTCC-3′; NQO1, F: 5′-GGCAGAAGAGCACTGATCGTA-3′, R: 5′-TGATGGGATTGAAGTTCATGGC-3′; Nrf2, F: 5′-TACTCCCAGGTTGCCCACA-3′, R: 5′-CATCTACAAACGGGAATGTCTGC-3′; GAPDH, F: 5′-TCGGAGTCAACGGATTTGGT-3′, R: 5′-TGGAATTTGCCATGGGTGGA-3′. Assays were repeated three times and the 2^−ΔΔ Cq^ method was used to calculate the relative abundance of mRNA.

### 4.5. Western Blot Analysis

Nuclear and cytoplasmic proteins were extracted using a nuclear and cytoplasmic protein extraction kit (Beyotime, Shanghai, China) according to the manufacturer’s instructions. Whole cell proteins were extracted as follows. Cells were harvested and sonicated in RIPA buffer containing protease inhibitor cocktail (Bimake, Shanghai, China). Then, lysates were centrifuged at 10,000× *g* at 4 °C for 15 min. After collecting supernatant, protein concentrations were determined by ultra-micro spectrophotometer (Implen, Munich, Germany). Proteins (30–50 μg) were separated on 10% sodium dodecyl sulfate-polyacrylamide gel electrophoresis (SDS-PAGE) and transferred onto polyvinylidene difluoride (PVDF) membranes. Membranes were blocked with 5% non-fat milk for 1–2 h at room temperature and incubated with primary antibodies of Nrf2, Keap1, HO-1, NQO1, GCLC, GCLM, Ub, Lamin B, and α-tubulin overnight at 4 °C. After being washed by Tris-buffered saline and Tween 20 (TBST), membranes were incubated with secondary antibodies for 1–2 h at room temperature and then detected with a chemiluminescence imaging system (Tanon 5500, Shanghai, China). Protein levels were standardized by comparison with Lamin B or α-tubulin. Representative blots were chosen from three independent experiments.

### 4.6. Immunofluorescence Assay

HepG2 cells (4 × 10^4^/well) were cultured on coverslips in 24-well plates for 24 h and subsequently treated with 20 μM of fisetin for 6 h. After treatment, cells were fixed using 4% paraformaldehyde solution (Dingguo, Beijing, China) for 40 min, permeabilized with 0.1% Triton-X (Solarbio, Beijing, China) for 20 min and blocked with goat serum (Boster, Wuhan, China) for 60 min. Then, cells were incubated overnight with Nrf2 primary antibody at 4 °C. Then, cells were incubated with fluorescent secondary antibody for 2 h at room temperature and stained with DAPI for 5 min. Finally, coverslips were mounted using antifade mounting medium (Boster, Wuhan, China) on slides, observed, and photographed under a fluorescence microscope (ZEISS, Jena, Germany).

### 4.7. Dual-Luciferase Reporter Gene Assay

A dual-luciferase reporter assay was used to determine promoter activity in transiently transfected cells. HepG2 cells (2 × 10^5^ cells/well) were plated in 6-well plates and cultured overnight in RPMI 1640 medium containing 10% FBS in an incubator. Cells were then transfected with pARE-luc plasmid (10 μL/well) and lipofectamin 3000 transfection reagent from Thermo Fisher (St. Louis, MO, USA) according to the manufacturer’s instructions. After being transfected with pARE-luc plasmid, cells were treated with different concentrations of fisetin (0, 10, 20, and 30 μM) for 24 h. Luciferase activities were detected using a dual-luciferase assay kit following the manufacturer’s instructions. Relative luciferase activity was calculated according to the relative light unit (RLU) of the firefly luciferase divided by the RLU of the renilla luciferase. The AREs of pARE-luc are as follows:

5′-GACTGAGGGTGACTCAGCAAAATCACTGAGGGTGACTCAGCAAAATC-3′
3′-CTGACTCCCACTGAGTCGTTTTAGTGACTCCCACTGAGTCGTTTTAG-5′.

### 4.8. Immunoprecipitation

After treatment with fisetin, HepG2 cells were lysed with cell lysis buffer containing 1 mM PMSF. The lysates were stirred at 4 °C for 2 h, and the homogenates were centrifuged at 12,000× *g* for 15 min at 4 °C. The supernatants were collected, and protein concentrations were determined by ultra-micro spectrophotometer. For immunoprecipitation, the cell extracts (0.5 mg) were pre-incubated with 2 μg of normal rabbit IgG and 20 μL of Protein A agarose beads for 2 h to reduce non-specific reactions. The mixture was centrifuged at 4 °C at 2500 rpm for 5 min. The supernatants were then incubated overnight with 2 μg of anti-Nrf2 antibody at 4 °C and then mixed with 30 μL of Protein A agarose beads at 4 °C for 3 h. Immunoprecipitation solutions were then centrifuged at 2500 rpm for 5 min at 4 °C to collect the beads, and the beads were washed once with cell lysis buffer. The complexes were eluted with 30 μL of SDS loading buffer, heated at 100 °C for 5 min, and subsequently analyzed by western blotting.

### 4.9. Statistical Analysis

The experimental results were expressed as the mean ± SD of at least three independent experiments. Analyses were performed with SPSS 18.0 (Chicago, IL, USA). Statistically significant differences were calculated with Student’s *t* test between two groups and one-way ANOVA among multiple groups for data satisfying assumptions of normal distribution and homogeneity of variance. Otherwise, data were analyzed with non-parametric tests. *P* < 0.05 was considered statistically significant. 

## 5. Conclusions

This study demonstrates that fisetin enhances the level of Nrf2 by prolonging the half-life and inhibiting the ubiquitination degradation of Nrf2. These actions finally result in Nrf2 accumulating in the nucleus to mediate ARE-driven antioxidant enzymes such as HO-1 expression to eliminate ROS caused by oxidative stress. Our data suggest that fisetin is an important antioxidant bioactive compound and deserves intensive scientific exploration for the prevention and treatment of oxidative stress-related diseases.
